# Probiotics and Gastrointestinal Infections

**DOI:** 10.1155/2008/290769

**Published:** 2009-02-04

**Authors:** Robert A. Britton, James Versalovic

**Affiliations:** ^1^Department of Microbiology and Molecular Genetics, Michigan State University, East Lansing, MI 48824, USA; ^2^Departments of Pathology, Baylor College of Medicine and Texas Children's Hospital, 6621 Fannin Street, MC 1-2261, Houston, TX 77030, USA

## Abstract

Gastrointestinal infections are a major cause of morbidity and mortality worldwide, particularly in developing countries. The use of probiotics to prevent and treat a variety of diarrheal diseases has gained favor in recent years. Examples where probiotics have positively impacted gastroenteritis will be highlighted. However, the overall efficacy of these treatments and the mechanisms by which probiotics ameliorate gastrointestinal infections are mostly unknown. We will discuss possible mechanisms by which probiotics could have a beneficial impact by enhancing the prevention or treatment of diarrheal diseases.

## 1. INTRODUCTION

Within the microbiota, individual bacteria containing important genes may benefit the host in different ways. As one considers the vast community of commensal microbes, subsets of these organisms may have important physiologic benefits for the host in the context of human nutrition and host:microbe interactions. Probiotics may stimulate immunity, regulate immune signaling pathways, produce antipathogenic factors, or induce the host to produce antipathogenic factors. Probiotics may produce secreted factors that stimulate or suppress cytokines and cell-mediated immunity. These factors may also interfere with key immune signaling pathways such as the NF-*κ*B and MAP kinase cascades. Probiotics may produce factors that inhibit pathogens and other commensal bacteria, effectively enabling these microbes to compete effectively for nutrients in complex communities. Microbes that produce antipathogenic factors may represent sources of novel classes of antimicrobial compounds, and these factors may be regulated by master regulatory genes in particular classes of bacteria. Microbes can also regulate signaling pathways in immune cells that result in the production of antimicrobial factors by mammalian cells, effectively resulting in remodeling of intestinal communities and prevention or treatment of infections.

Gastrointestinal infections are a major cause of morbidity and mortality worldwide. Studies conducted in 2006 found that, globally, severe diarrhea and dehydration are responsible each year for the death of 1,575,000 children under the age of five. This represents 15% of the 10.5 million deaths per year of children in this age group [1]. According to recent estimates, acute gastroenteritis causes as many as 770,000 hospitalizations per year in the United States [2]. Enteric pathogens include viruses (rotaviruses, noroviruses) and bacteria such as different strains of pathogenic *Escherichia coli*, toxigenic *Clostridium difficile*, *Campylobacter jejuni*, and *Vibrio cholerae*. These pathogens produce different types of toxins that can cause severe or life-threatening dehydration and diarrhea. Despite medical advances in diagnosis and treatment, the percent and number of hospitalized pediatric patients less than 5 years of age with severe rotavirus infection significantly increased when a recent time period (2001–2003) was compared to an earlier time period (1993–1995) [3]. In addition to the typical pattern of acute gastroenteritis, infectious agents such as enteropathogenic *E. coli* (EPEC) may cause persistent, chronic diarrhea in children lasting longer than 1 week [4]. Such persistent infections may increase the risk of dehydration and long-term morbidities. Importantly, the relative contributions of EPEC and other bacterial pathogens to disease remains controversial to some extent. A recent study highlighted that increased relative risk of gastrointestinal disease in children was only demonstrable for enteric viruses [5].

Recent studies have highlighted long-term morbidities associated with gastroenteritis. Early childhood diarrhea predisposes children to lasting disabilities, including impaired fitness, stunted growth, and impaired cognition and school performance [6]. Along with this data, new research on maternal and child undernutrition reported in *The Lancet* in January 2008 links poor nutrition with an increased risk for enteric infections in children. Furthermore, irritable bowel syndrome (IBS), a costly and difficult to treat condition that affects 20% of the United States population [7], has medical costs of up to $30 billion per year, excluding prescription and over-the-counter drug costs [8]. IBS is precipitated by an episode of acute gastroenteritis in up to 30% of all cases in prior studies [9]. Therefore, preventing or treating acute gastroenteritis before long-term sequelae develop would drastically reduce hospitalizations, disability-adjusted life years, and both direct and indirect medical costs.

Accurate diagnosis of acute gastroenteritis is an ongoing challenge even in sophisticated academic medical centers. In a pediatric patient population exceeding 4,700 children, less than 50% of stool samples that underwent complete microbiologic evaluation yielded a specific diagnosis [10]. Enteric viruses represented the predominant etiologic agents in acute gastroenteritis in children less than 3 years of age, and bacteria caused the majority of cases of acute gastroenteritis in children older than 3 years of age [10]. The diagnostic challenges with enteric viruses include the relative paucity of stool-based molecular or viral antigen tests and the inability to readily culture most enteric viruses. Bacterial pathogens may be difficult to identify (such as most strains of disease-causing *E. coli*) because of the lack of specific assays for these infections. The relative insensitivity of stool-based toxin assays for the detection of toxigenic *C. difficile* precludes accurate diagnosis. In a children's hospital setting, combination toxin antigen testing yielded sensitivity below 40% in pediatric patients (J. Versalovic, unpublished data). The introduction of new molecular assays for real-time PCR detection of toxin genes directly in stool has markedly improved the ability to diagnose antimicrobial-associated diarrhea and colitis due to toxigenic *C. difficile* [11]. In addition, approximately 15–25% of cases of antimicrobial-associated diarrhea are caused by *C. difficile*. The prevalence of antimicrobial-associated diarrhea and gastrointestinal disease highlights the importance of alternatives to antibiotic strategies for treatment. Furthermore, antibiotics have limited utility for the treatment of gastroenteritis in general. Antimicrobial agents are not generally recommended as prevention strategies because of the problems of antibiotic resistance and antimicrobial-associated disease. Thus, instead of suppressing bacterial populations with antibiotics, can probiotics be used to remodel or shift microbial communities to a healthy state [12]?

## 2. PROBIOTICS

### 2.1. The need for mechanistic details of probiotic action

The use of probiotics to prevent and treat a wide variety of conditions has gained favor in the past decade. This is in part due to a need to find alternatives to traditional therapies such as antibiotics as well as the lack of good treatments for GI ailments. While there are increasing reports of the efficacy of probiotics in the treatment of diseases such as pouchitis [13, 14], diarrhea [15–17], and irritable bowel syndrome [18], the scientific basis for the use of probiotics is just beginning to be understood. We will focus on the potential applications for probiotics in the treatment of diarrheal disease. Several examples will highlight how probiotics may be selected for and utilized against pathogens causing gastroenteritis.

The concept of using probiotic microorganisms to prevent and treat a variety of human ailments has been around for more than 100 years [19]. With the rise in the number of multidrug resistant pathogens and the recognition of the role that the human microbiota plays in health and disease, a recent expansion in the interest in probiotics has been generated. This phenomenon is apparent in both the numbers of probiotic products being marketed to consumers as well as the increased amount of scientific research occurring in probiotics. Although many of the mechanisms by which probiotics benefit human beings remain unclear, probiotic bacteria are being utilized more commonly to treat specific diseases.

Several definitions of what constitutes a “probiotic” in the literature have been formulated. For this review, we use the definition derived in 2001 by the Food and Agricultural Organization (FAO) and the World Health Organization (WHO)—“Probiotics are live microorganisms which when administered in adequate amount confer a health benefit on the host.” [20]. This definition is the currently accepted definition by the International Scientific Association for Probiotics and Prebiotics (ISAPP) (http://www.isapp.net/).

### 2.2. Antipathogenic activities

**Figure 1:**
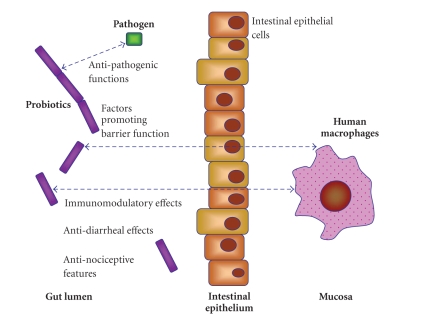
*Probiotics and Beneficial Effects in the Intestine*. Depiction of the interactions between beneficial bacteria (left side), their secreted factors, pathogens, and the intestinal mucosa (right side). Potential beneficial effects of probiotics are listed. Only two host cell types are shown, intestinal epithelial cells and macrophages although other cell types including dendritic cells, lymphocytes, myofibroblasts, and neutrophils comprise the intestinal mucosa. The arrows indicate the release and possible distribution of secreted factors derived from probiotics.

Perhaps the most important scientific question regarding the use of probiotics in medicine is the identification of mechanisms by which probiotics impact human health. Several mechanisms have been implicated but most have not been experimentally proven (Figure 1). Here, we discuss possible mechanisms that are relevant for the treatment of diarrheal diseases. We will highlight research examples that support these putative mechanisms whenever possible.

### 2.3. Stimulation of host antimicrobial defenses

Many probiotics have been shown to produce antipathogenic compounds ranging from small molecules to bioactive antimicrobial peptides. Most of these studies have focused on the in vitro susceptibility of pathogens to products secreted by probiotic bacteria. In most cases, the ability of an antimicrobial compound secreted by a probiotic organism to inhibit the growth of a pathogen in vivo has not been demonstrated. Conceptually, an antimicrobial compound produced by an organism would need to be produced at a high enough level and in the right location in the intestinal tract to exert a strong effect on a pathogen in vivo.

An elegant proof of principle for direct action of a probiotic-produced antimicrobial against a pathogen was recently reported by Corr et al. who demonstrated that production of the bacteriocin Abp118 by *Lactobacillus salivarius* was sufficient to protect mice from disease by infection with *Listeria monocytogenes* [21]. To prove the action of the bacteriocin was directly responsible for the protection of the mice, they generated a *L. salivarius* strain that was unable to produce Abp118 and showed that this mutant was incapable of protecting against *L. monocytogenes* infection. Notably, they were able to express a gene that confers immunity to the Abp118 bacteriocin within *L. monocytogenes* and showed that this strain was now resistant to the probiotic effect of *L. salivarius* within the mouse. This study provided clear evidence that a probiotic-derived bacteriocin could function directly on a pathogen in vivo.

### 2.4. Pathogen exclusion via indirect mechanisms

In addition to producing antimicrobial compounds that act directly on pathogens, probiotics may stimulate host antimicrobial defense pathways. The intestinal tract has a number of mechanisms for resisting the effects of pathogens including the production of defensins [22]. Defensins are cationic antimicrobial peptides that are produced in a number of cell types including Paneth cells in the crypts of the small intestine and intestinal epithelial cells. A deficiency in alpha-defensin production has been correlated with ileal Crohn's disease [23, 24]. Tissue samples from patients with Crohn's disease showed a lower level of alpha-defensin production and extracts from these samples exhibited a reduced ability to inhibit bacterial growth in vitro. Moreover, some pathogenic bacteria have evolved mechanisms to inhibit the production or mechanism of action of defensins (reviewed in [25]).

Probiotics may act to stimulate defensin activity via at least two mechanisms. First, probiotics may stimulate the synthesis of defensin expression. This has been demonstrated for human beta defensin 2 (hBD-2), whose expression is upregulated by the presence of several probiotic bacteria via the transcription factor NF-*κ*B [26, 27]. The implication is that probiotic strains with this capability would strengthen intestinal defenses by increasing defensin levels. This effect is also observed with certain pathogenic bacteria and thus is not a specific property of probiotic bacteria. Second, many defensins are produced in a propeptide form that must be activated via the action of proteases. One well-characterized example is the activation of the murine defensin cryptdin (an alpha-defensin that is produced by Paneth cells) by the action of matrix metalloprotease 7 (MMP-7) [28]. Mice defective for MMP-7 are more susceptible to killing by *Salmonella*. Evidence indicates that bacteria can stimulate the production of MMP-7 in the intestine [29]. Thus, one mechanism in which probiotics could participate in activating defensins is by stimulating the production of MMPs in the intestinal tract. Alternatively, probiotics could produce proteases that themselves activate defensins in the intestinal lumen. Although there is no evidence yet to support this mechanism, a subset of lactobacilli and streptococci encode MMP-like proteins in their genomes (R. Britton, unpublished observation). These MMPs are not found in any other bacteria and thus it will be interesting to determine what effect they have on host cell function.

### 2.5. Immunomodulation

Rather than directly inhibiting the growth or viability of the pathogen, probiotics may compete for an ecological niche or, otherwise, create conditions that are unfavorable for the pathogen to take hold in the intestinal tract. There are many possible mechanisms for how pathogen exclusion may take place. First, several probiotics have been demonstrated to alter the ability of pathogens to adhere to or invade colonic epithelial cells in vitro, for example, see [30, 31]. Second, probiotics could sequester essential nutrients from invading pathogens and impair their colonization ability. Third, probiotics may alter the gene expression program of pathogens in such a way as to inhibit the expression of virulence functions [32]. Lastly, probiotics may create an unfavorable environment for pathogen colonization by altering pH, the mucus layer, and other factors in the local surroundings. It is important to note that although many of these possible effects have been demonstrated in vitro, the ability of probiotics to exclude pathogens in vivo remains to be proven.

### 2.6. Enhancing intestinal barrier function

Probiotics may have strain-dependent effects on the immune system. Different strains representing different *Lactobacillus* species demonstrated contrasting effects with respect to proinflammatory cytokine production by murine bone marrow-derived dendritic cells [33]. Specific probiotic strains counteracted the immunostimulatory effects of other strains so that probiotics have the potential to yield additive or antagonistic results. Interestingly, in this study, the anti-inflammatory cytokine IL-10 was maintained at similar levels [31]. Different probiotic *Lactobacillus* strains of the same species may also yield contrasting effects with respect to immunomodulation. Human breast milk-derived *Lactobacillus reuteri* strains either stimulated the key proinflammatory cytokine, human tumor necrosis factor (TNF), or suppressed its production by human myeloid cells [34]. The mechanisms of action may be due, not surprisingly, to contrasting effects on key signaling pathways in mammalian cells. Probiotic strains such as *Lactobacillus rhamnosus* GG (LGG) may activate NF-*κ*B and the signal transducer and activator of transcription (STAT) signaling pathways in human macrophages [35]. In contrast, probiotic *Lactobacillus* strains may suppress NF-*κ*B signaling [36, 37] or MAP kinase-/c-Jun-mediated signaling [34]. Stimulation of key signaling pathways and enhancement of proinflammatory cytokine production may be important to “prime” the immune system for defense against gastrointestinal infections. Conversely, suppression of immune signaling may be an important mechanism to promote homeostasis and tolerance to microbial communities with many potential antigens, and these immunosuppressive functions may promote healing or resolution of infections.

### 2.7. Why understanding mechanisms is important?

The disruption of epithelial barrier function and loss of tight junction formation in the intestinal epithelium may contribute to pathophysiology and diarrheal symptoms observed during infection with certain pathogens [38, 39]. Loss of tight junctions can lead to increased paracellular transport that can result in fluid loss and pathogen invasion of the submucosa. Pathogens may secrete factors such as enterotoxins that may promote excessive apoptosis or necrosis of intestinal epithelial cells, thereby disrupting the intestinal barrier. Enteric pathogens may also cause effacing lesions at the mucosal surface due to direct adherence with intestinal epithelial cells (e.g., EPEC). In contrast, probiotics have been reported to promote tight junction formation and intestinal barrier function [40, 41]. Although the mechanisms of promoting barrier integrity are not well understood, probiotics may counteract the disruption of the intestinal epithelial barrier despite the presence of pathogens. Probiotics may also suppress toxin production or interfere with the abilities of specific pathogens to adhere directly to the intestinal surface. As a result, pathogens may have a diminished ability to disrupt intestinal barrier function.

### 2.8. Important considerations for the use of probiotics: strain selection and microbial physiology

An important challenge in the field of probiotics is the identification of genes and mechanisms responsible for the beneficial functions exerted by these microbes. Successful identification of mechanistic details for how probiotics function will have at least three important benefits. First, understanding mechanisms of action will provide a scientific basis for the beneficial effects provided by specific microbes. These breakthrough investigations will help move probiotics from the status of dietary supplements to therapeutics. Second, understanding mechanisms of probiosis and the gene products produced by probiotics will allow for the identification of more potent probiotics or the development of bioengineered therapeutics. As an example, the anti-inflammatory cytokine IL-10 was postulated to be a potential therapeutic for the treatment of inflammatory bowel disease. To test this hypothesis, a strain of *Lactococcus lactis* engineered to produce and secrete IL-10 was constructed and demonstrated to reduce colitis in a murine model [42]. Early clinical trials in patients with inflammatory bowel disease indicate some relief from symptoms when treated with the IL-10 overproducing strain. Third, the identification of gene products that are responsible for ameliorating disease will allow researchers, industry, and clinicians to follow the production of these products as important biomarkers during probiotic preparation. As discussed below, the physiological state of microbes can be crucial to the functions of probiotics. Thus, it will be important to be able to follow the production of important bioactive molecules when culturing and processing probiotics for applications in animals and humans.

### 2.9. Probiotics and diarrhea

Probiotics are considered to be living or viable microorganisms by definition. Unlike small molecules that are stable entities, probiotics are dynamic microorganisms and will change gene expression patterns when exposed to different environmental conditions. This reality has two important implications for those who choose to use these organisms to combat human or animal diseases. First, probiosis is a strain-specific phenomenon. As defining a bacterial species is challenging in this age of full genome sequencing, it is clear that probiotic effects observed in vitro and in vivo are strain specific. For example, modulation of TNF production by strains of *Lactobacillus reuteri* identified strains that were immunostimulatory, immunoneutral, and immunosuppressive for TNF production [34, 43]. These findings highlight the strain-specific nature of probiotic effects exerted by bacteria. Thus, it is important for research groups and industry to be cautious with strain handling and tracking so that inclusion of correct strains is verified prior to administration in clinical trials.

The second key point is that the physiology of the probiotic strain is an important consideration. Being live microorganisms, the proteins and secondary metabolites that are being produced will change depending on growth phase. This feature raises a number of important issues for the stability and efficacy of probiotic strains. First, probiotics are subjected to numerous environmental stresses during production and after ingestion by the host. Most notably, probiotics used to treat intestinal ailments or whose mode of action is thought to be exerted in the intestinal tract must be able to survive both acid and bile stress during transit through the gut. The physiological state of the microbe is an important characteristic that determines whether cells will be susceptible to different types of environmental stress [44, 45]. For example, exponentially growing cells of *L. reuteri* are much more susceptible to killing by bile salts than cells in stationary phase [45]. Thus, it is important to consider the physiological state of the cells in terms of stress adaptation not only for survival in the host but also during production. Second, the expression of bioactive molecules, which are most often responsible for the health benefits exerted by probiotics, is often growth phase-dependent. For example, our groups have been investigating the production of immunomodulatory compounds and antimicrobial agents by strains of *L. reuteri*. In both cases, these compounds are more highly expressed in the entry into and during stationary phase (unpublished observation).

## 3. PROBIOTICS AND THE PREVENTION AND TREATMENT OF GASTROENTERITIS—EXAMPLES

Commensal-derived probiotic bacteria have been implicated as therapy for a range of digestive diseases, including antibiotic-associated colitis, *Helicobacter pylori* gastritis, and traveler's diarrhea [46]. Probiotic formulations may include single strains or combinations of strains. *L. reuteri* is indigenous to the human gastrointestinal tract, is widely present in mammals, and has never been shown to cause disease. In human trials, probiotic treatment with *L. reuteri* in small children with rotaviral gastroenteritis reduced the duration of disease and facilitated patient recovery [15, 16], while in another study, it prevented diarrhea in infants [17]. Despite the promising data from clinical trials, the primary molecular mechanisms underlying the antipathogenic properties of *L. reuteri* remain unknown.

Probiotics may be effective for the prevention or treatment of infectious gastroenteritis. In the context of disease prevention, several studies with different probiotic strains have documented that these bacteria may reduce the incidence of acute diarrhea by 15–75% depending on the study [17, 47–50]. Although the relative impacts on disease incidence vary depending on the specific probiotic strain and patient population, consistent benefits for disease prevention have been demonstrated in multiple clinical studies. In one disease prevention study [49], supplementation with *Bifidobacterium lactis* significantly reduced the incidence of acute diarrhea and rotavirus shedding in infants. Studies that examined potential benefits of probiotics for preventing antimicrobial-associated diarrhea have yielded mixed results [51–54]. One prevention study reported a reduction in incidence of antimicrobial-associated diarrhea in infants by 48% [52].

Probiotics may also be incorporated in treatment regimens for infectious gastroenteritis. Several meta-analyses of numerous clinical trials with different probiotics documented reductions in disease course of gastroenteritis that ranged from 17 to 30 hours [49, 50, 55]. Examined another way, meta-analyses of probiotics used in clinical trials of gastroenteritis noted significant reductions of incidence of diarrhea lasting longer than 3 days (prolonged diarrhea). The incidence of prolonged diarrhea was diminished by 30% or 60%, respectively, depending on the study [50, 56] (summarized in [55]). The probiotic agent, LGG, contributed to a significant reduction in rotavirus diarrhea by 3 days of treatment when administered to children as part of oral rehydration therapy [57]. Recent data compilations of a large series of probiotics trials by the Cochrane Database of Systematic Reviews (http://www.cochrane.org/) have yielded promising conclusions. As of 2008, probiotics appear to be effective for preventing acute gastroenteritis in children and may reduce duration of acute disease. Additionally, probiotics are promising agents for preventing and treating antimicrobial-associated diarrhea, although intention-to-treat analyses have not demonstrated benefits.

### 3.1. Clostridium difficile and antibiotic-associated diarrhea

In what follows, we highlight some possible mechanisms by which probiotics can be used to ameliorate gastroenteritis. Because a number of infectious agents cause diarrhea, colitis, and gastroenteritis, we will only focus on a few examples with the idea that many of the mechanisms discussed can be extended to other bacterial or viral causes of diarrhea.

#### 3.1.1. The potential role of probiotics in treating CDAD

An estimated 500,000–3,000,000 cases of *Clostridium difficile-*associated diarrhea (CDAD) occur annually with related health care costs exceeding $1 billion per year [58–60]. CDAD occurs primarily in patients that have undergone antibiotic therapy in a health care setting, indicating that alterations in the intestinal microbiota are important for the initiation of CDAD. In a small but increasing number of cases, more severe complications will occur including pseudomembranous colitis and toxic megacolon. Moreover, the emergence of metronidazole-resistant strains of *C. difficile* has diminished the efficacy of metronidazole, and vancomycin- and metronidazole-induced cecitis reinforces the need for new therapies for the treatment and prevention of CDAD [61, 62].

Approximately 10–40% of patients treated for an initial bout of CDAD will show recurrent disease, often with multiple episodes [63]. Such recurrences are often refractory to existing therapies including antibiotic therapy. Patients with recurrent CDAD had a marked decrease in the diversity of organisms in their fecal microbiota while patients that were free of recurrent disease had a normal microbiota [64]. Thus, therapies that restore a normal microbiota or suppress *C. difficile* growth while allowing the repopulation of the intestine with a favorable microbiota may be important to resolve infections and maintain intestinal health.

#### 3.1.2. Eradication of *C. difficile* through the production of antimicrobial compounds

Probiotic organisms have been used to treat recurrent *C. difficile* in the past and in a few cases have showed a modest effect in ameliorating recurrent disease [63]. This application has been somewhat controversial and at this time the use of probiotics in ameliorating CDAD is not recommended [65]. However, the organisms tested were not specifically isolated for the treatment of CDAD and, therefore, may have not been the appropriate strains to be used to prevent recurrent CDAD. In what follows, we outline potential mechanisms in which carefully selected or engineered probiotics could be used in the treatment of *C. difficile* and the eradication of this pathogen.

#### 3.1.3. Competitive exclusion of *C. difficile* using probiotics

CDAD is currently treated by the use of antimicrobial agents that are effective against *C. difficile*, most often vancomycin or metranidazole. Because these drugs are broad-spectrum antibiotics, they likely play a role in recurrent disease by suppressing the normal intestinal microbiota. Using antimicrobial compounds that target *C. difficile* while allowing restoration of resident organisms would be one possible mechanism to prevent recurrent CDAD.

#### 3.1.4. Probiotics and *C. difficile* spore germination

As mentioned above, CDAD is usually an infection that is acquired in the hospital or other health care setting.

Therefore, a probiotic that could competitively exclude *C. difficile* could be administered prior to entry into the hospital. Unfortunately, little is known about how and where *C. difficile* colonizes the intestine. Once this information is known, strategies for blocking colonization with probiotics can be developed.

Nonetheless, a promising probiotic approach using non-toxigenic *C. difficile* has been described. Using a hamster model of *C. difficile* infection, Gerding et al. demonstrated a protective effect of populating the hamster with strains of *C. difficile* that are unable to produce toxin prior to challenge with a virulent toxin-producing strain [66]. Colonization of the intestinal tract by the nontoxigenic strain appeared to be required for protection. Currently, this probiotic approach is under investigation for use in humans (http://www.viropharma.com/).

### 3.2. Enterohemorrhagic *E. coli*

A likely contributor to the difficulty in eradicating *C. difficile* from the intestine is the ability of the organism to develop stress-resistant spores. The identification of probiotic strains that can prevent either spore formation or the germination of spores in the intestinal tract provides a promising avenue to combat CDAD. Recent work on spore germination has provided in vitro assays in which inhibitory activities of probiotics can be tested [67].

Germination of spores in the laboratory requires the presence of bile acids, with taurocholate and cholate demonstrating the best activity [67]. Thus, bile acids could play a role in signaling to *C. difficile* that spores are in the correct location of the gut to germinate. Sorg and Sonenshein have recently proposed a mechanism by which the reduction in the intestinal microbiota could lead to efficient spore germination and overgrowth of *C. difficile* [67]. They found that the bile acid deoxycholate (DOC) was able to induce spore germination but that subsequent growth was inhibited due to toxic effects of DOC on vegetative *C. difficile*. Their work suggests a model in which a reduction in the concentration of DOC in the intestine, due to the disruption of the normal microbiota, removes this key inhibitor of *C. difficile* growth. DOC is a secondary bile acid produced from dehydroxylation of cholate by the enzyme 7*α*-dehydroxylase, an activity that is produced by members of the intestinal microbiota. While it is unclear whether or not antibiotic therapy reduces the level of DOC in the intestine, it is tempting to speculate that providing probiotic bacteria capable of producing 7*α*-dehydroxylase may prevent intestinal overgrowth by *C. difficile* while the normal microbiota is being reestablished.

#### 3.2.1. Toxin sequestration and removal

Enterohemorrhagic *E. coli* (EHEC) infections cause sporadic outbreaks of hemorrhagic colitis throughout the world (∼100,000 cases per year in the United States) [68]. Most infections result in the development of bloody diarrhea but a subset (∼5–10%) of EHEC patients (mostly children) will develop the life-threatening condition hemolytic uremic syndrome (HUS) [69, 70]. HUS is the leading cause of kidney failure in children. EHEC, which likely evolved from an EPEC strain [71], also produces attaching and effacing lesions on host epithelial cells and reduces intestinal epithelial barrier function. In addition, EHEC strains are characterized by the expression of Shiga toxin (Stx) genes, and thus they can be labeled as Shiga-toxin-producing *E. coli* (STEC). Currently, only supportive therapy for EHEC infection is available since antibiotic therapy may increase the risk of developing HUS, and therefore, novel therapies must be developed. One promising alternative therapeutic may be the use of probiotics to treat EHEC infections.

#### 3.2.2. Inhibition of toxin production by EHEC—identification of strains that repress the lytic functions of lambda

Shiga toxins are ribosome-inactivating proteins that inhibit protein synthesis by removing a specific adenine residue from the 28S rRNA of the large ribosomal subunit [72]. Shiga toxin is required for the development of HUS and recent work has indicated that EHEC strains mutated for Shiga toxin production fail to cause disease in a germfree mouse model [73]. Indeed, injection of Shiga toxin with LPS directly into mice is sufficient to generate a HUS-like disease in the kidneys of mice [74]. Therefore, Shiga toxin is an important mediator of HUS and therapies aimed at neutralizing its activity are expected to reduce or eliminate this life-threatening complication although current attempts at Shiga toxin neutralization have been unsuccessful [75].

As a possible mechanism for treating EHEC disease and reducing the incidence of HUS cases, Paton et al. have generated “designer probiotics” in which the oligosaccharide receptor (Gb_3_) for Stx is expressed on the cell surface of an *E. coli* strain [76–78]. This probiotic strain was shown to be capable of neutralizing Stx in vitro. As a proof-of-concept, mice that were challenged with a STEC strain were protected by administration of the probiotic expressing the Gb_3_ receptor [79]. The protective effect was observed even when the strains were formalin-killed prior to use, supporting the hypothesis that toxin sequestration and removal was the mechanism by which the mice were protected. Similar results have been obtained using bacteria-expressing receptors for toxins produced by other diarrheal pathogens including enterotoxigenic *E. coli* (most common cause of traveler's diarrhea) and *Vibrio cholerae*.

#### 3.2.3. Inhibition of pathogen adherence and strengthening of intestinal barrier functions

Stx genes are carried on lambdoid prophages and are usually located in a late transcribed region of the virus, near the lytic genes [80]. Since no mechanism for toxin secretion has been identified, the location of Stx near the lytic genes suggests that phage activation and cell lysis are responsible for Stx production and release. This genetic juxtaposition suggests that therapeutics that suppress the lytic decision of lambda in vivo would greatly reduce or eliminate complications caused by systemic release of Stx.

### 3.3. Rotavirus

A key interaction of EHEC, as well as EPEC, with the intestinal epithelium is the formation of attaching and effacing lesions on the surface of the epithelium [81]. This interaction is brought about by factors secreted directly from the bacterium into the host cell, where a redistribution of the actin cytoskeleton occurs. EHEC and EPEC infection also induces a loss of tight junction formation and reduction of the intestinal epithelial barrier by inducing the rearrangement of key tight junction proteins including occludin [82, 83]. Therapies that would either disrupt this interaction of EHEC/EPEC with the intestinal epithelium or inhibit the loss of barrier function should ameliorate disease.

Probiotics have shown some success inhibiting adhesion, A/E lesion formation and enhancing barrier function in response to EHEC infection in vitro. Johnson-Henry et al. tested the ability of *Lactobacillus rhamnosus* GG to prevent loss of barrier integrity and formation of A/E lesions induced by EHEC infection of cell culture in vitro [40]. They found that pretreatment of intestinal epithelial cells in vitro with LGG was sufficient to reduce the number of A/E lesions and to prevent loss of barrier function as measured by transepithelial resistance, localization of tight junction proteins, and barrier permeability assays. Importantly, live LGG was required for these effects as heat-killed bacteria were not effective in preventing EHEC effects on epithelial cells.

Enteric viruses including noroviruses and rotavirus represent major causes of gastroenteritis, especially in young children. Rotavirus infection results in acute gastroenteritis with accompanying dehydration and vomiting mainly in children 3–24 months of age. Human rotavirus primarily infects intestinal epithelial cells of the distal small intestine, resulting in enterotoxin-mediated damage to intestinal barrier function. Recent studies indicate that probiotics may reduce the duration and ameliorate disease due to rotavirus infection ([84]; G. Preidis and J. Versalovic, unpublished data). Probiotics promoted intestinal immunoglobulin production and appeared to reduce the severity of intestinal lesions due to rotavirus infection in a mouse model. These findings and related investigations suggest that probiotics may diminish the severity and duration of gastrointestinal infections by mechanisms independent of direct pathogen antagonism. Probiotics may also promote healing and homeostasis by modulating cytokine production and facilitating intestinal barrier function.

## 4. CONCLUDING REMARKS

Probiotics may provide an important strategy for the prevention and treatment of gastrointestinal infections. Specific bacteria derived from human microbial communities may have key features that establish these microbes as primary candidates for probiotic therapies. These beneficial microbes may have different effects within the host such as prevention of pathogen proliferation and function. Probiotics may also stimulate the host's immune function and mucosal barrier integrity. By working via different mechanisms of probiosis, probiotics may yield effects at different steps in the process. Probiotics may prevent disease from occurring when administered prophylactically. Probiotics may also suppress or diminish severity or duration of disease in the context of treatment. As our knowledge of the human microbiome advances, rational selection of probiotics based on known mechanisms of action and mechanisms of disease will facilitate optimization of strategies in therapeutic microbiology. Ultimately, we expect that probiotics will help to promote stable, diverse, and beneficial microbial communities that enhance human health and prevent disease.
